# Understanding the impact of early onset colorectal cancer on quality of life: a qualitative analysis of online forum data

**DOI:** 10.1007/s11136-024-03857-z

**Published:** 2024-11-26

**Authors:** Alice Spencer, Christopher Bedding, Emma Nicklin, Hélène Flint, Alexandra Gilbert

**Affiliations:** 1 Leeds Institute of Medical Research at St James’ Hospital, University of Leeds, Leeds, UK; 2 Co-researcher, Hertfordshire, UK

**Keywords:** Colorectal cancer, Early onset, Online forums, Adolescent, Young adult, Quality of life

## Abstract

**Purpose:**

Early onset colorectal cancer (EOCRC) is rising. The profile of health-related quality of life (HRQOL) impacts may differ in this younger cohort. Online forums are a source of unfiltered information regarding patient experience. This study used a qualitative analysis of online forum messages to elicit the unique HRQOL impacts of EOCRC.

**Methods:**

Messages were extracted from an online EOCRC UK forum. Inductive coding (with 10% dual-coding) and thematic analysis were used to describe the impact of diagnosis and treatment on HRQOL.

**Results:**

Data extraction and analyses were performed over one month; 463 messages (dated 01/04/2019 to 31/03/2024) were included. There was 100% concordance on dual-coding for main themes. Eight themes emerged: (1) diagnostic pathway and barriers; (2) parenthood and effect on children; (3) employment and finances; (4) fertility and early menopause; (5) stoma implications; (6) support systems, relationships and isolation; (7) sport and exercise and (8) mental health.

**Conclusions:**

Qualitative thematic analysis of online forum data is a novel and efficient methodology for understanding the impact of cancer on HRQOL. Identified themes overlapped with those published in previous systematic reviews. This study offers new insights into the impact of isolation, early menopause, benefits of parenthood, psychological impact on children and practical and psychological implications of potential infertility in EOCRC. Current understanding of the diagnostic challenges and unique HRQOL impacts of EOCRC raises future research questions regarding how colorectal cancer services should evolve to provide support more in keeping with the needs of this growing younger cohort.

**Supplementary Information:**

The online version contains supplementary material available at 10.1007/s11136-024-03857-z.

## Introduction

Colorectal cancer is the third most common cancer worldwide, responsible for 9.6% of all cancer diagnoses in 2022 [1]. There has been a shift in the burden of colorectal cancer towards younger patients, particularly in high income countries [2, 3]. One in five colorectal cancer diagnoses in the United States now occur in patients under 55 [2]. Across Europe the rising incidence of early onset colorectal cancer (EOCRC), referring to diagnosis before age 50, demonstrates a birth cohort phenomenon. The most sustained increases in incidence are seen in individuals aged 20–29 suggesting the burden of EOCRC will increase as this younger population ages [4, 5]. EOCRC may be a distinct disease entity to colorectal cancer diagnosed after 50 with some unique risk factors [6–8] and differences in histological subtype, molecular abnormalities and site of disease being reported [6, 9]. Additionally, individuals under 50 are more likely to be diagnosed at a later stage than older counterparts [6, 9–11], potentially reflecting a combination of poor awareness of EOCRC [12], diagnostic pathway delays [13] and more aggressive disease subtypes in younger patients [6, 7].

By virtue of diagnosis at a different stage in life, patients with EOCRC may experience differing health-related quality of life (HRQOL) impacts and be disproportionately affected in areas such as employment and finances, social and family functioning, body image, intimate relationships and fertility compared to older counterparts [14–19]. These impacts may be felt long after treatment is completed, as age at diagnosis has been shown to be an independent determinant of colorectal cancer survivorship experience [20].

Despite the rising incidence of EOCRC and likely unique HRQOL impacts, a recent systematic review of HRQOL impacts in EOCRC concluded that the psychosocial impacts are poorly investigated [17]. Understanding these impacts is vital for development of supportive interventions for this growing patient cohort and qualitative research has a vital role in exploring this emerging topic [21]. Online forums provide a space for cancer patients and their families to seek support, share information and discuss their care [22]. Such forums are a rich source of patient-generated data and provide researchers and clinicians an unfiltered description of patient lived experience [23]. Qualitative analysis of forum messages can offer insight into cancer patient experience of diagnosis, treatment and survivorship [24–30]. Previous research has utilised various qualitative methodologies to describe the challenges and unmet needs of patients with EOCRC [15, 29, 31], and report on specific aspects of the cancer journey such as self-advocacy [26] and diagnostic barriers in primary care [25]. This study aims to build on this existing body of research.

Utilising this novel methodology of online forum analysis, we aim to explore issues surrounding the diagnostic pathway and the range of specific HRQOL issues pertinent to individuals with EOCRC.

## Methods

A phenomenological approach was employed as we aimed to explore patient experience of a specified phenomenon, namely living with EOCRC [32]. Comprehensive guidance on performing internet-mediated research was followed [23].

### Nature of the forum

The forum was chosen as it was a popular forum with many users, written in English and contained a large amount of publicly available data in the form of user-generated title threads to which related messages could be added. Crucially, within its hierarchical structure, it had a designated board for discussion of issues related to colorectal cancer in individuals under 50 from which all data was extracted.

### Data extraction and analysis

**Fig. 1:**
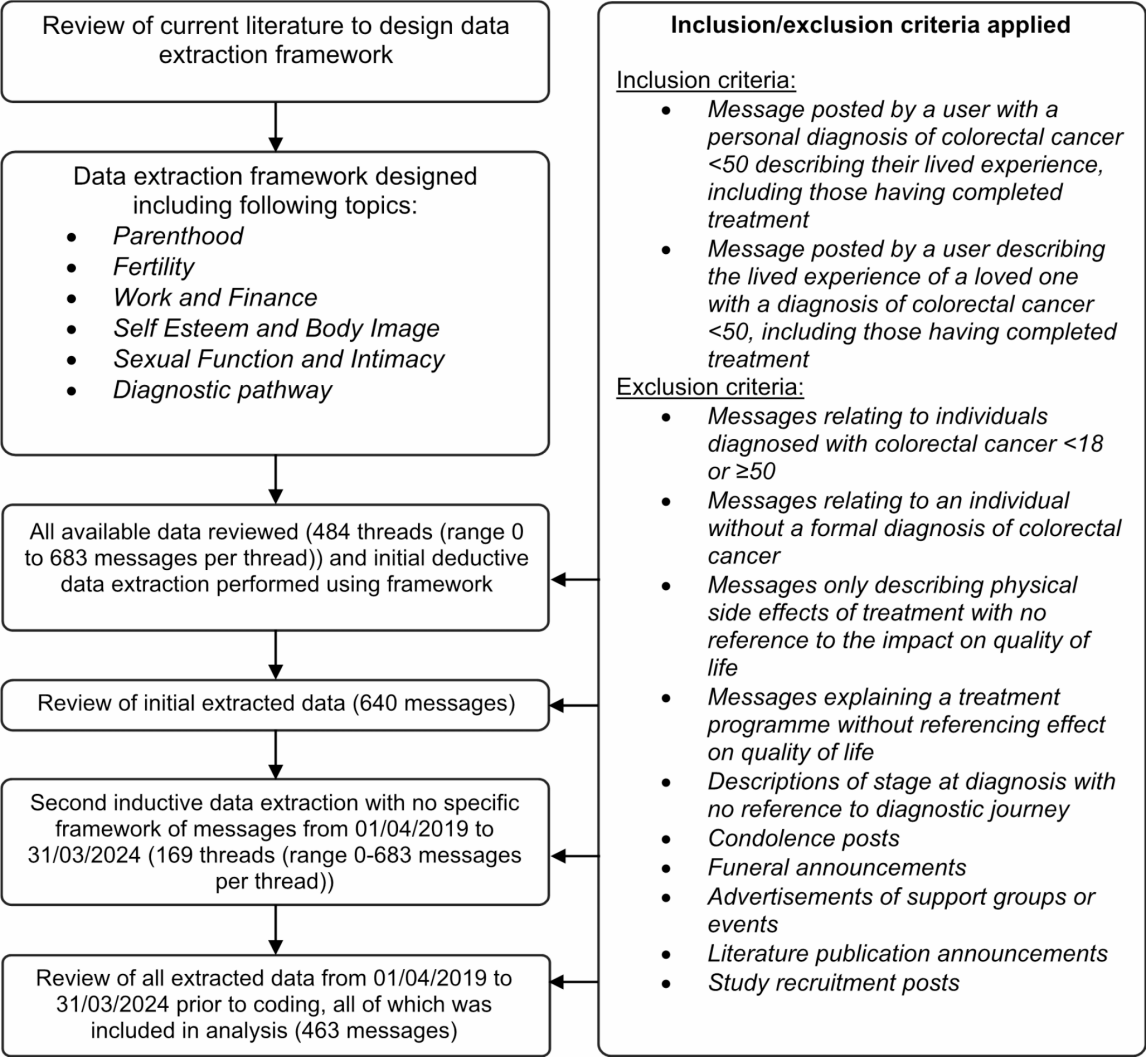
Process for data extraction and inclusion/exclusion criteria

Data refers to the content of title threads or messages. An iterative approach to data extraction was applied with inclusion/exclusion criteria for message sampling outlined in Fig. 1. Following initial review of the forum a decision was made to exclude messages which only described physical side effects of treatment with no reference to other aspects of HRQOL, as these were unlikely unique to younger patients. Initial deductive extraction enabled early data familiarisation, confirmation of sufficient available data to meet the study aim and estimation of the quantity of messages required to reach theoretical saturation [21, 33]. As such, further extraction and analysis was limited to messages ordered over the last five years (01/04/2019 to 31/03/2024). Repeat *inductive* extraction was paramount to the phenomenological approach, ensuring all potential issues emerged from the data without restrictions of a preconceived framework [32].

Coding and thematic analysis was conducted based on guidance published by Braun and Clarke [33, 34] and Saunders et al. [21] and performed using Microsoft Excel [35]. An inductive and iterative approach was applied to codebook generation and assigning codes. To ensure consistency all messages were coded by the primary researcher (AS: researcher clinician in oncology, female), with 10% dual-coding by a co-researcher (CB: postgraduate researcher, specialist in psycho-oncology and patient centred outcomes, male) to ensure concordance and agreed code definitions. Generating themes and subthemes was an iterative process; initial themes were drafted from the codebook, reviewed and refined, then discussed within the research team to ensure they accurately reflected the data. Content analysis of codes was not used to determine themes but provided confidence of theoretical saturation and a tool to practise researcher reflexivity, ensuring undue weight was not applied to a single particularly relatable message or code. A co-researcher with lived experience of EOCRC assisted in analysis through interpretation and appropriate coding of messages and final agreement of themes, offering a vital patient perspective.

### Ethical considerations

Comprehensive ethical guidance regarding internet-mediated research was followed [23, 36]. Ethical approval was granted by University of Leeds Research Ethics Committee. Forum owners granted approval to utilise data. The data was in the public domain so user consent was not required, but careful consideration was given to protecting anonymity and confidentiality of posting users [23, 36]. All data was anonymised on extraction and longer published quotes were paraphrased and reverse searched to ensure no traceability. All paraphrased quotes were reviewed by co-researchers and modified if needed ensuring meaning was retained.

## Results

Following data extraction (Figs. 1), 463 messages from 111 threads were included in analysis, 83% (*n* = 383) were posted by individuals with a personal diagnosis of EOCRC, 14% (*n* = 67) by family or friends, 3% (*n* = 13) undetermined. Agreement was achieved on dual-coding (100% for main themes) and codebook definitions.

**Fig. 2:**
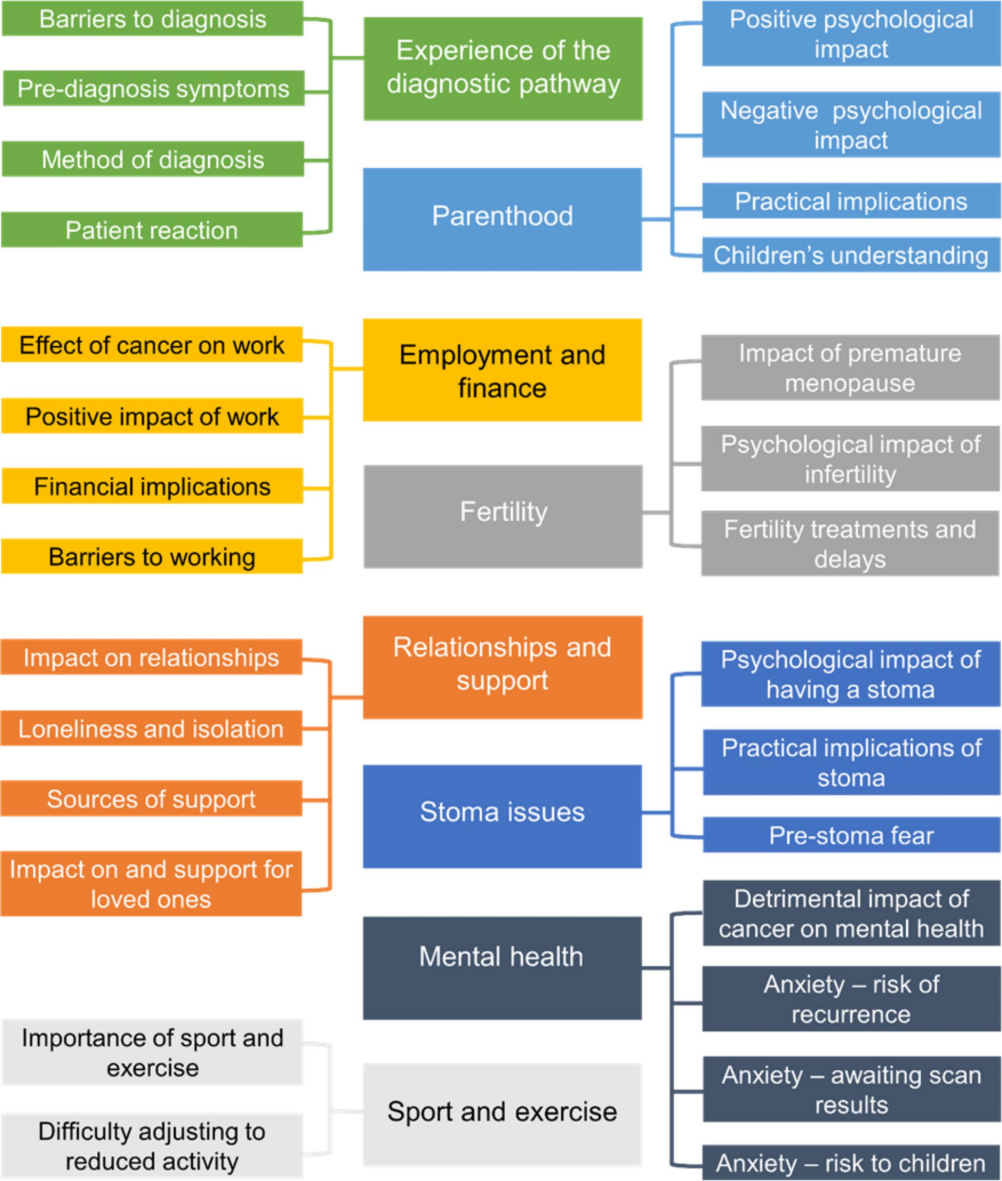
Map of main themes and corresponding subthemes

Eight overarching themes with relevant subthemes were constructed (Fig. 2). Supportive quotes are shown in Table 1.

**Table 1:** Supportive quotes for themes. *Denotes paraphrased quotes to protect user anonymity, all were reviewed by co-researchers ensuring meaning was accurately retained

Quote number	Overarching theme	Quote
**1**	Experience of the diagnostic pathway	“At my age it was a big shock…I thought I was a healthy, young person to suddenly having bowel cancer”*
**2**	Experience of the diagnostic pathway	“Getting past your GP and making someone listen can be the biggest hurdle”*
**3**	Experience of the diagnostic pathway	“My partner was finally diagnosed at a late stage after months of trying to get answers from their doctors”*
**4**	Parenthood	“I need to be here for my children so I’m focussed on giving this my all”*
**5**	Parenthood	“I love spending time with my kids - they help me stay positive”*
**6**	Parenthood	“I only focus on my cancer when I have to…other times I’m pre-occupied with work and being a parent”*
**7**	Parenthood	“Thinking about my children not having a father is so heartbreaking”*
**8**	Parenthood	“Fears that I may not see my children grow up and have their own families are overwhelming”*
**9**	Employment and finance	“At our age its unfair - particularly the effect on our careers which haven’t even really begun”*
**10**	Employment and finance	“I will have to work as much as possible as I don’t get sick pay”*
**11**	Fertility	“I’ve been told about the risk of infection from egg harvesting but I feel like I can’t risk delaying my chemo. It feels like I don’t have long to make lots of decisions”*
**12**	Stoma issues	“Accepting my stoma was really difficult, I can only accept it as I know I would not be alive without it”*
**13**	Relationships and support	“I am finding it really hard as there are not many people my age going through the same experience as me”*
**14**	Relationships and support	“Some of my friends disappeared when they found out about my diagnosis”*
**15**	Sport and exercise	“I’ve been swimming and working out when I’m able to and it makes a massive difference”*
**16**	Mental health	“The main thing for me has been the impact on my mental health”*
**17**	Mental health	“I hope to have many more years of life left so 5 years surveillance doesn’t feel like enough”*

### Experience of the diagnostic pathway

Bleeding, pain, anaemia and change in bowel habit were the most commonly described pre-diagnosis symptoms. Interestingly, some users stated they had “no symptoms” but within the same message described symptoms clinically attributable to colorectal cancer. Most described diagnosis via their general practitioner, but a considerable number referenced attending accident and emergency and/or having emergency surgery. Some chose to access private healthcare for their diagnosis as the “faster route”. Many were shocked by their diagnosis (Table 1, quote 1), some were angry.

Many experienced barriers to diagnosis in both primary and secondary care (quote 2), feeling dismissed or “fobbed off” by healthcare providers due to their age and/or being misdiagnosed.

Some described ignoring symptoms and delayed presentation, but many stressed a need for multiple presentations to healthcare services or strong self-advocacy to access tests or referrals (quote 3).

### Parenthood

Identifying as a parent was a strong subtheme. Number and ages of children were emphasised in introduction statements. Many described the positive impact of parenthood on the cancer journey. Children were a strong motivating factor in treatment (quote 4), a source of happiness (quote 5) and parental responsibilities were a welcome distraction (quote 6).

Parenthood created additional psychological burden. Parental “guilt”, “worry” about the effects of treatment on childcare abilities, and anxieties and sadness exacerbated by thoughts of children “having to grow up without [them]” were described (quotes 7&8).

Parental duties were often adapted with users “planning activities around [treatment]” or loved ones shouldering a greater proportion of parental duties. Children’s reactions to their parent’s diagnosis were described, and users identified how “hard” it is for younger children “to understand”. Users emphasised the positive impact of dedicated support services for children and used the forum to offer advice or direction towards charitable organisations providing this.

### Employment and finance

Many identified the positive impact of employment, describing returning to work as “helpful”, providing a “welcome distraction” and a “sense of normality and structure”. For one user, stopping work long-term was “unimaginable”. Some referenced use of employer provided insurance to access private healthcare. The impact of cancer on work was regularly discussed, many described undertaking adapted work roles, mostly “phased return” or “flexible” working. Most changes described were not long-term, but some took “early retirement” or reduced to part-time. Some described frustration at the diagnosis halting their careers (quote 9).

Changes to work roles were due to physical side effects of treatment, “exhaustion” and a “shift in mentality”. Many referenced a “supportive work environment” but some described feeling “forgotten” by employers. Financial pressures were referenced, particularly for self-employed users (quote 10).

### Fertility

Subthemes differed between sexes. The psychological impact of potential infertility was described more commonly in females, one described feeling “devastated”. Female users referenced fertility preservation delaying cancer treatment and described decision-making surrounding this as an additional stressor during an already stressful time (quote 11). This was referenced once in relation to male fertility by a patient’s partner.

Male patients shared positive experiences of successful pregnancies post-treatment. Information giving from clinicians about fertility implications was generally clear, but one partner described mixed messages from healthcare professionals about the potential impact on male fertility.

Female users identified issues relating to menopausal symptoms and impact on bone health. The important role of general practitioners in menopause management and providing information was stressed. In contrast to fertility information, users discussed poor information giving, having to “do your own research” and clinicians focussing on cancer cure without focus on “overall health”.

### Stoma issues

Many expressed a negative view of their stoma. For some this was strongly emotive; messages described feeling “pre-occupied”, “distraught” and a “hatred” for their stoma, others described minimal inconvenience, but they would “rather not have it”. Pre-stoma fear or apprehension was an issue, some felt “terrified” by the prospect. Despite this, many achieved “acceptance” of their stoma, commonly due to learning to see their stoma as “lifesaving” (quote 12). For the majority, appearance changes due to stoma, weight loss, hair loss and rashes carried minimal or no psychological burden, but some described reduced self-esteem and for one patient this was “traumatic”. Having to deal with the practical implications of a stoma was a commonly discussed issue with users providing peer to peer advice or advocating for certain services and resources.

### Relationships and support

Users utilised a range of informal and formal support structures via family and friends, medical teams and charitable organisations. Despite this, many described feeling lonely or isolated. For the majority, this was due to struggling to find others who could “relate” to their experience or difficulty talking “honestly” with loved ones rather than geographical barriers or COVID restrictions (quote 13). One user described changing after cancer - becoming “stronger” and “wiser” - and family members having difficulty processing that change. Users stressed the value of the forum in combatting these feelings and advocated for other support groups they had attended.

Some experienced loss of friendships after diagnosis (quote 14), and one described breakdown of a romantic relationship. Interestingly, only one message referenced impact on sexual intimacy or function.

Individuals identified the psychological impact on loved ones and need for support mechanisms for them. Some felt a “burden” to their families or partners and discussed the challenge of dealing with their changing familial role or balancing caring responsibilities for older parents.

### Sport and exercise

Many users referenced exercising and playing sport as an established part of their pre-cancer life and getting “back to sports/exercise” was a priority. For some this represented getting “back to normal” or feeling “like something [they] can control”. A reduction in fitness was described as a “big adjustment”, and the impact on normal exercise routines represented the physical impacts of cancer and treatment. Many described the benefits of sport and exercise to their overall wellbeing during their cancer journey (quote 15), particularly in relation to mental wellbeing. Emphasising the value placed on sport and exercise, one user praised a privately funded supported return to exercise programme.

### Mental health

The psychological impact of cancer and treatment was apparent in relation to all previously described themes, however the detrimental impact of cancer on overall mental health also emerged as an additional individual theme. For some, the mental health impact was one of the most challenging aspects of their cancer journey (quote 16).

Generalised anxiety, fear, and anxiety relating to waiting for scan results - “scanxiety” – were frequently described. Anxieties more specific to a younger population also emerged, such as anxiety surrounding risk to children and anxiety relating to surveillance and recurrence which may be more pertinent for younger patients who, by virtue of diagnosis earlier in life, will have longer life expectancy after curative treatment (quote 17).

## Discussion

Using online forum analysis this study highlighted a range of HRQOL issues amongst individuals with EOCRC. These impacts overlap with those reported in a recent systematic review of the HRQOL impacts of EOCRC which included 14 quantitative studies and one qualitative interview study [14, 17]. Waddell et al. [17] identified career and financial impacts, body image, intimate relationships, sexual dysfunction, emotional distress (including anxiety), social functioning and effect on personal relationships and family functioning (including caring for children) as important issues, all of which are encompassed within the themes identified in this study. Notably, sexual dysfunction and impact on intimate relationships were rarely identified in messages. This likely reflects individual’s reluctance to discuss such private issues within an open internet forum, as sexual dysfunction in adolescent and young adult (AYA) cancer survivors [37, 38] and colorectal cancer patients is widely reported [31, 39–41]. More traditional methodologies may be required to explore these domains.

Except for stoma issues, many of the impacts identified in this study are not disease-specific and are applicable to a range of cancers in AYAs [42, 43]. Accordingly, the HRQOL impacts reported here overlap with eight of nine overarching domains identified in a systematic review of HRQOL issues facing AYAs with cancer, which included 58 quantitative studies and 11 qualitative utilising interviews [18]. The domain not reflected in our study was impact on cognitive function which is potentially an issue individuals may not volunteer without direct questioning or disclose in an open forum.

The overlap with impacts identified through systematic review both specifically for EOCRC [17] and AYA cancers [18] support our findings and highlight the utility of this novel methodology of using forum data to identify HRQOL issues. Furthermore, this study offers new insights into the HRQOL issues in EOCRC not reported by Waddell et al. [17] including the specific impacts of isolation and early menopause. We also expand on the impact of parenthood - particularly the positive benefit of parenthood and psychological impact on children – and describe the psychological impact of potential infertility and practical implications of fertility treatments.

In addition to describing the practical impacts of cancer on child-caring abilities, we identified the psychological impacts on children and demand for tailored children’s support. A mixed methods study of unmet needs in EOCRC also reported individuals unable to find such services [31]. Emotional and behavioural problems in children of parents with cancer and the positive impact of adaptive coping strategies have also been highlighted, supporting a drive for dedicated children’s services [44]. Providing intensive interventions for all families is not practically possible but our findings show some already utilise resources provided by charitable organisations and advocate the use of these to peers within forum messages. Understanding existing resources and signposting appropriately may be an impactful first step for clinicians in providing support. Further research is needed to investigate how to identify families most likely to benefit from more intensive interventions.

Themes of isolation and difficulties relating to family, friends and other cancer patients by virtue of diagnosis at a young age, are echoed in other studies [29, 31]. Whilst it is not surprising that the positive impact of connecting with other EOCRC patients is highlighted in a study analysing data from a forum specifically designed for this purpose, the value of interactions with other EOCRC survivors as a source of support is emphasised in studies using more traditional qualitative methodologies [15]. Sodergren et al. [18] describe isolation amongst AYA patients and highlight a study of patients with acute lymphoblastic leukaemia (ALL) reporting the benefit of opportunities to make new connections with fellow patients [45]. Compared to ALL which is usually diagnosed in younger people [46], the typical colorectal cancer patient is still over 50, despite the rising incidence of early onset disease [2]. Therefore, the opportunities for EOCRC patients to makes links with others going through the same situation may be more limited, and the need for access to dedicated support groups to forge these connections may be especially pertinent amongst EOCRC patients.

The impact of infertility after treatment for EOCRC was frequently discussed. Whilst risk of gonadal failure is reportedly low following adjuvant chemotherapy for colon cancer [47, 48], gonadal failure post pelvic irradiation for rectal cancer is an expected side effect [47, 49, 50]. Fertility preservation guidelines recommend sperm cryopreservation for males, and embryo or oocyte cryopreservation and/or ovarian transposition pre-radiotherapy in females, with a caveat of variable success rates in the latter [50, 51]. Discussions about fertility and referrals to reproductive specialists are recommended to occur as early as possible [51]. Users described waits for fertility treatments leading to cancer treatment delays, and the additional stress of decision-making surrounding this. Other studies report a lack healthcare provider recommendations for post-treatment fertility issues [15]. This may reflect a knowledge gap and lack of established fertility preservation referral pathways in colorectal cancer services initially designed to treat older patients. Recommendations state all practitioners involved in treating young cancer patients should be able to address these issues [51]. Improvements likely require education of healthcare professional to assist in complex decision making and facilitate fertility discussions early in the cancer journey.

Concerning post-treatment menopause, which follows pelvic irradiation [52], some users reported poor information giving. A lack of regard for addressing menopausal symptoms and consequences, such as osteoporosis and cardiovascular disease, due to prioritisation of cancer surveillance is also reported in the literature and the importance of multidisciplinary team management with a vital role for primary care providers is emphasised [52]. Ensuring good communication between specialist cancer services and primary care may be the most important step in ensuring adequate follow-up for menopause issues.

Regarding the diagnostic pathway, a considerable number of emergency diagnoses was not an unexpected finding. An observational study of EOCRC diagnoses in England found younger patients were more commonly diagnosed as an emergency and less likely to be diagnosed via two-week wait than older counter parts (*p* < 0.001) [13]. The perception of barriers to diagnosis in both primary and secondary care is reflected in existing literature and age bias affecting clinical assessment is a common theme [15, 25, 29, 53]. Guilt associated with overburdening strained secondary care services and age-related guideline constraints have been highlighted as barriers for general practitioner’s in referring suspected EOCRC [53]. In August 2023, UK referral guidance was updated to include wider use of faecal immunochemical testing, offering an easily accessible quantitative measure for risk stratifying patients in primary care to enable better prioritisation of secondary care investigations, which may assist in overcoming these barriers [5, 54, 55]. The impact of these guideline changes and campaigns to increase awareness of EOCRC [12] is an important future research question.

The key strength of this work is the use of forum data to reveal key HRQOL impacts of EOCRC. Using this time and cost-efficient methodology, this study captured the issues described through systematic review of existing literature [17] and identified additional impacts. It also provided additional qualitative content which is comparatively lacking within this literature. Analysis of online forum data offers unique advantages over traditional qualitative methodologies. All content is user-generated with no interaction between researcher and participant, eliminating the potential influence of interviewer bias or restrictions of an interview structure and enabling emergence of unexpected themes [23]. This is supported by the emergence of multiple topics of interest through repeat inductive extraction. Users described difficulty talking openly and relating to others given their relatively uncommon diagnosis but stressed the value of the forum in facilitating honest discussion. Forum analysis likely enabled exposure of important experiences such as feelings of isolation which may not have been discussed in interviews. The efficiency of this methodology in terms of time and cost savings is a major strength. Use of forum data allowed a vast quantity of easy to access data, from many individuals, available from the public domain to be retrieved and analysed at reduced cost, with no need for participant recruitment, lengthy ethics delays or conduction and transcription of interviews as required in other studies [14, 15, 23, 31]. This enabled the research to be conducted over a few months, rather than a longer time period usually required for qualitative methodologies, whilst still maintaining high quality data outcomes and encapsulating a significantly greater number of views than is usually possible with qualitative work.

The inclusion of a co-researcher with lived experience in analysis of the results offered an invaluable perspective when interpreting forum message meaning. They highlighted the frequency with which messages offer peer-to-peer advice and advocated for existing services, which raises future research questions surrounding better utilisation of existing services along with designing of new interventions to tackle the HRQOL impacts in EOCRC. The concept of analysis of forum data to explore patient-to-patient advice giving statements has been explored in a recent study and presents further utility of this methodology [26].

This methodology has limitations. The study aims to analyse experiences of a specific group but cannot control characteristics of individuals posting within the forum. Where messages offered sufficient information, strict exclusion criteria were applied, and the forum board clearly stated it was a space for those with EOCRC. It is possible some messages included in analysis do not represent individuals with EOCRC, but this likely represents a very small proportion of analysed data. Potential sample bias with over-representation of certain genders, ethnicities or socio-economic groups must also be acknowledged. Project resource constraints precluded access to alternative analysis software or analysis of multiple forums, the forum used is registered to one country limiting cross-cultural validity. To facilitate analysis of sufficient data regarding HRQOL within the study timeframe, messages including symptom-only data were excluded however comparison of treatment and disease-related physical side-effects in early versus late onset disease presents an interesting separate research question as more aggressive treatment in younger patients is reported [9, 56]. Issues relating to sexual dysfunction, widely reported in existing literature [31, 37–41], were not revealed through forum analysis which may be an inappropriate methodology for exploring this sensitive issue. The ethical requirement to protect user anonymity requires data anonymisation and paraphrasing of published quotes, however all paraphrased quotes were reviewed by co-researchers ensuring meaning was retained. Data anonymisation precludes user tracking, meaning data stratification by demographics is not possible. One user describing the same experience in multiple messages represents multiple separate data points, as thematic rather than content analysis was applied this has less influence but should be considered in interpretation of the results.

## Conclusion

Individuals with EOCRC face barriers to diagnosis and experience HRQOL impacts which may not affect older patients traditionally diagnosed with colorectal cancer. Online forums provide a platform for patients to forge connections with others who can relate to their journey which may be more important for younger people who do not represent the typical colorectal cancer patient. Online forum analysis is a novel and efficient methodology for describing HRQOL using qualitative data which offers vital insights in research regarding patient experience and is comparatively lacking in current EOCRC literature [17]. Themes identified within this study overlap with those reported through systematic review [17, 18]. However, this study offers new insights into the impacts of isolation, early menopause, benefits of parenthood, need for supportive services for children and support surrounding fertility implications in EOCRC. Current understanding of the diagnostic challenges and unique HRQOL impacts of EOCRC raises important future research questions regarding how colorectal cancer services will need to evolve to provide support more in keeping with the needs of this growing younger cohort.

## Electronic supplementary material

Below is the link to the electronic supplementary material.

Supplementary Material 1
