# Correction: Understanding the impact of early onset colorectal cancer on quality of life: a qualitative analysis of online forum data

**DOI:** 10.1007/s11136-025-03938-7

**Published:** 2025-03-08

**Authors:** Alice Spencer, Christopher Bedding, Emma Nicklin, Hélène Flint, Alexandra Gilbert

**Affiliations:** 1 Leeds Institute of Medical Research at St James’ Hospital, University of Leeds, Leeds, UK; 2 Co-researcher, Hertfordshire, UK

**Correction to: Quality of Life Research**

10.1007/s11136-024-03857-z.

The abstract was missing from this article and should have read as below.

**Abstract**

**Purpose** Early onset colorectal cancer (EOCRC) is rising. The profile of health-related quality of life (HRQOL) impacts may differ in this younger cohort. Online forums are a source of unfiltered information regarding patient experience. This study used a qualitative analysis of online forum messages to elicit the unique HRQOL impacts of EOCRC.

**Methods** Messages were extracted from an online EOCRC UK forum. Inductive coding (with 10% dual-coding) and thematic analysis were used to describe the impact of diagnosis and treatment on HRQOL.

**Results** Data extraction and analyses were performed over one month; 463 messages (dated 01/04/2019 to 31/03/2024) were included. There was 100% concordance on dual-coding for main themes. Eight themes emerged: (1) diagnostic pathway and barriers; (2) parenthood and effect on children; (3) employment and finances; (4) fertility and early menopause; (5) stoma implications; (6) support systems, relationships and isolation; (7) sport and exercise and (8) mental health.

**Conclusions** Qualitative thematic analysis of online forum data is a novel and efficient methodology for understanding the impact of cancer on HRQOL. Identified themes overlapped with those published in previous systematic reviews. This study offers new insights into the impact of isolation, early menopause, benefits of parenthood, psychological impact on children and practical and psychological implications of potential infertility in EOCRC. Current understanding of the diagnostic challenges and unique HRQOL impacts of EOCRC raises future research questions regarding how colorectal cancer services should evolve to provide support more in keeping with the needs of this growing younger cohort.

The original article has been corrected.

